# Supplementary material for: Morphological changes and two Nodal paralogs drive left-right asymmetry in the squamate veiled chameleon (C. calyptratus)
Source: Front Cell Dev Biol. 2023 Apr 11;11:1132166. doi: 10.3389/fcell.2023.1132166 (PMC10126504; doi:10.3389/fcell.2023.1132166)
Supplement: Supplementary file 1 [file Image5.pdf]

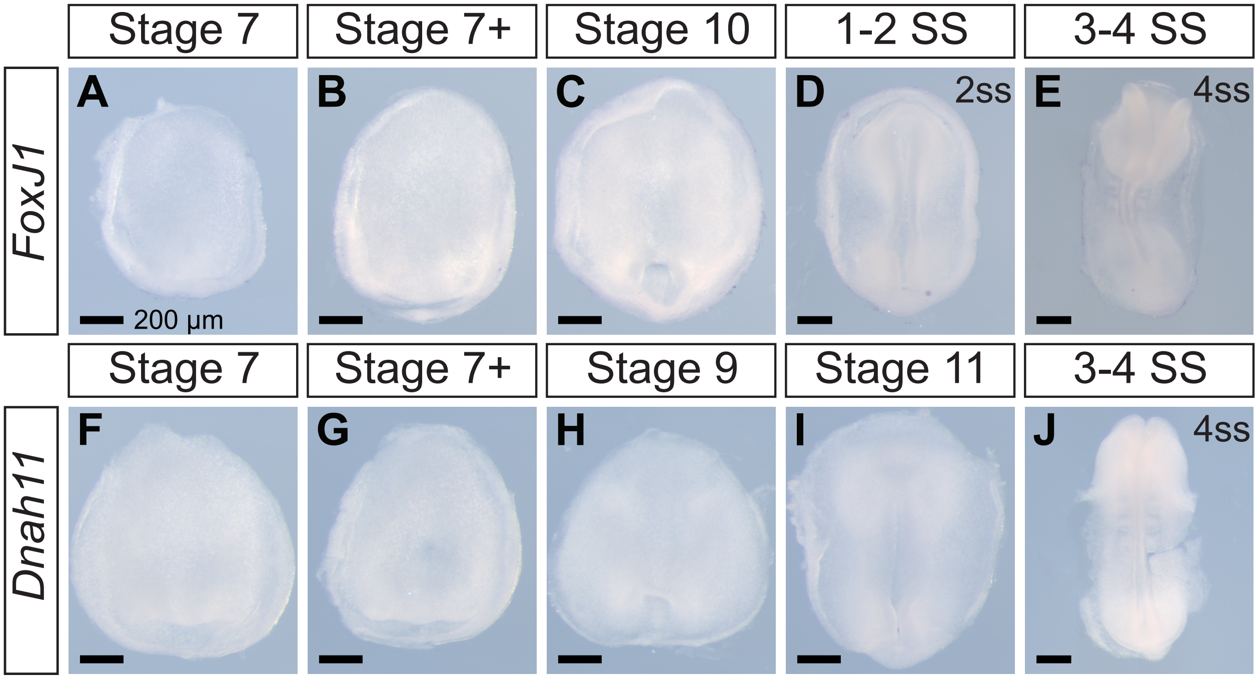

Supplementary Figure S5

Dorsal view of *FoxJ1* and *Dnah11* ciliary markers expression. All embryos are presented in dorsal view. Ventral view is available in Figure 4. **(A-E)** Whole mount RNA *in situ* hybridization for *FoxJ1* expression. **(F-J)** Whole mount RNA *in situ* hybridization for *Dnah11* expression. Scale bars are 200 µm.
