# Supplementary material for: Morphological changes and two Nodal paralogs drive left-right asymmetry in the squamate veiled chameleon (C. calyptratus)
Source: Front Cell Dev Biol. 2023 Apr 11;11:1132166. doi: 10.3389/fcell.2023.1132166 (PMC10126504; doi:10.3389/fcell.2023.1132166)
Supplement: Supplementary file 3 [file Image6.pdf]

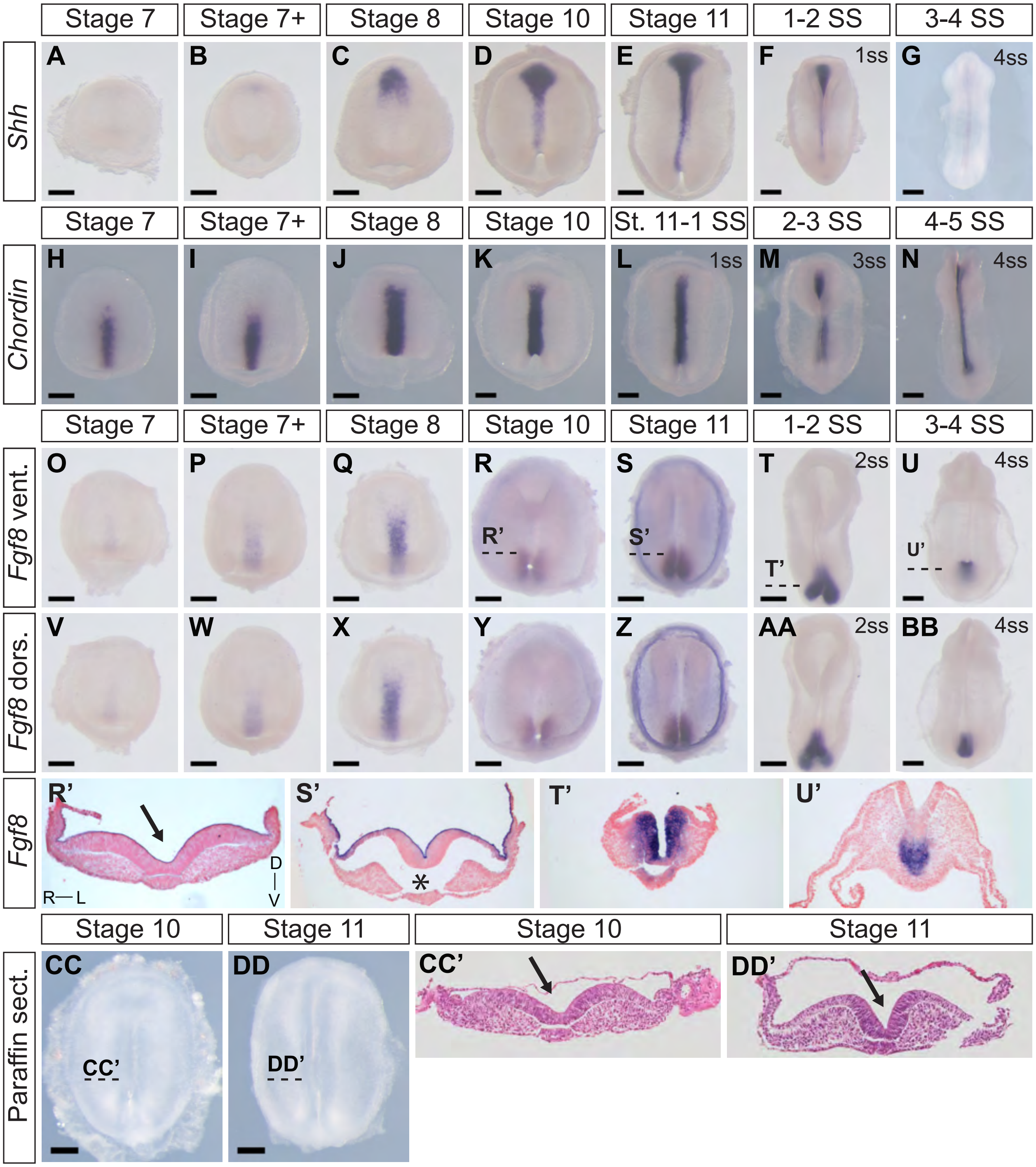

Supplementary Figure S6

Morphological changes in the embryo visualized through H&E staining, *Shh*, *Chordin* and *Fgf8* expression. **(A-N)** Dorsal view of embryos from Figure 6. **(A-G)** Whole mount RNA *in situ* hybridization for *Shh* expression. **(H-J)** Whole mount RNA *in situ* hybridization for *Chordin* expression. **(O-U)** Ventral view of whole mount RNA *in situ* hybridization for *Fgf8* expression. **(V-BB)** Dorsal view of embryos from **(O-U)**. **(R'-U')** Transverse sections of embryos from **(O-U)**, as indicated with dashed lines. Sections were counterstained with nuclear fast red for better visualization. **(CC-DD)** Ventral view of embryos, sectioned transversely and stained with H&E. Approximate location of sections indicated with dashed lines. **(CC'-DD')** Transverse paraffin sections of embryos in **(CC, DD)**, stained with H&E. Section orientation is as indicated in **(R')**. Arrows point to the medial hinge point of the neural tube. Asterisk reveals asymmetry in notochordal plate. All scale bars are 200 μm.
