# Supplementary material for: Morphological changes and two Nodal paralogs drive left-right asymmetry in the squamate veiled chameleon (C. calyptratus)
Source: Front Cell Dev Biol. 2023 Apr 11;11:1132166. doi: 10.3389/fcell.2023.1132166 (PMC10126504; doi:10.3389/fcell.2023.1132166)
Supplement: Supplementary file 4 [file Image4.pdf]

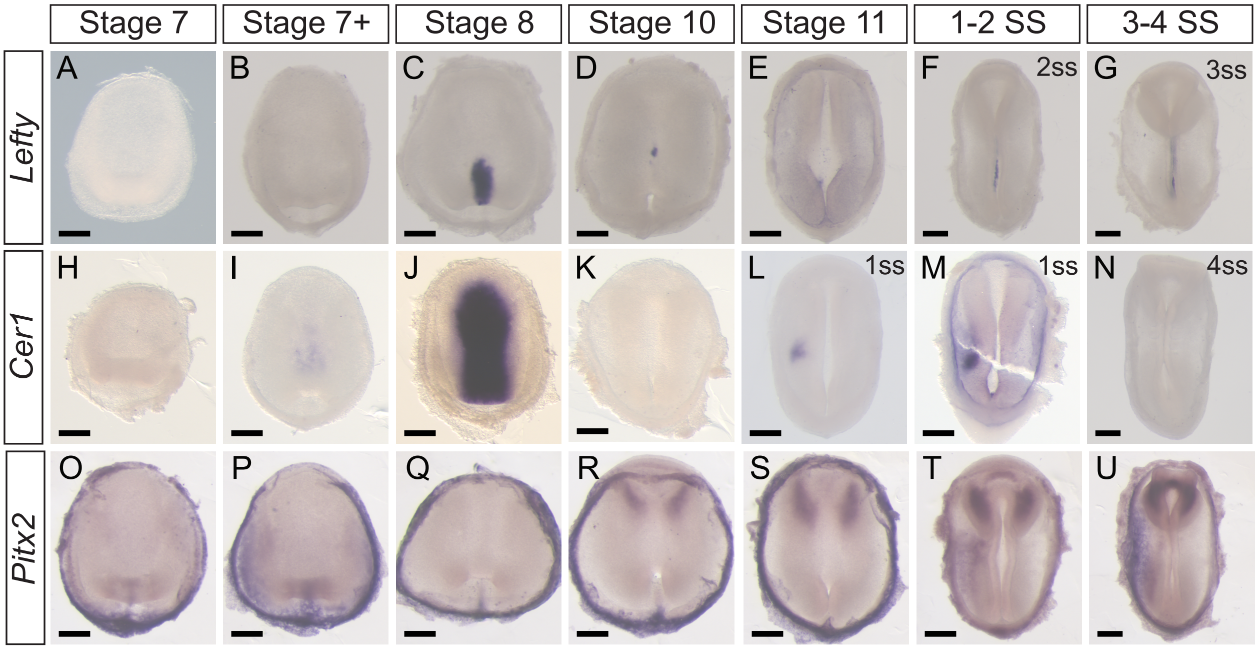

Supplementary Figure S4

Dorsal view of expression patters of key members of the Nodal cascade in veiled chameleon embryos. All embryos are presented in dorsal view. Ventral view is available in Figure 3. **(A-G)** Whole mount RNA *in situ* hybridization for *Lefty* expression. **(H-N)** Whole mount RNA *in situ* hybridization for *Cer1* expression. **(O-U)** Whole mount RNA in situ hybridization for *Pitx2* expression. All scale bars are 200 μm.
