# Supplementary material for: Morphological changes and two Nodal paralogs drive left-right asymmetry in the squamate veiled chameleon (C. calyptratus)
Source: Front Cell Dev Biol. 2023 Apr 11;11:1132166. doi: 10.3389/fcell.2023.1132166 (PMC10126504; doi:10.3389/fcell.2023.1132166)
Supplement: Supplementary file 5 [file Image2.pdf]

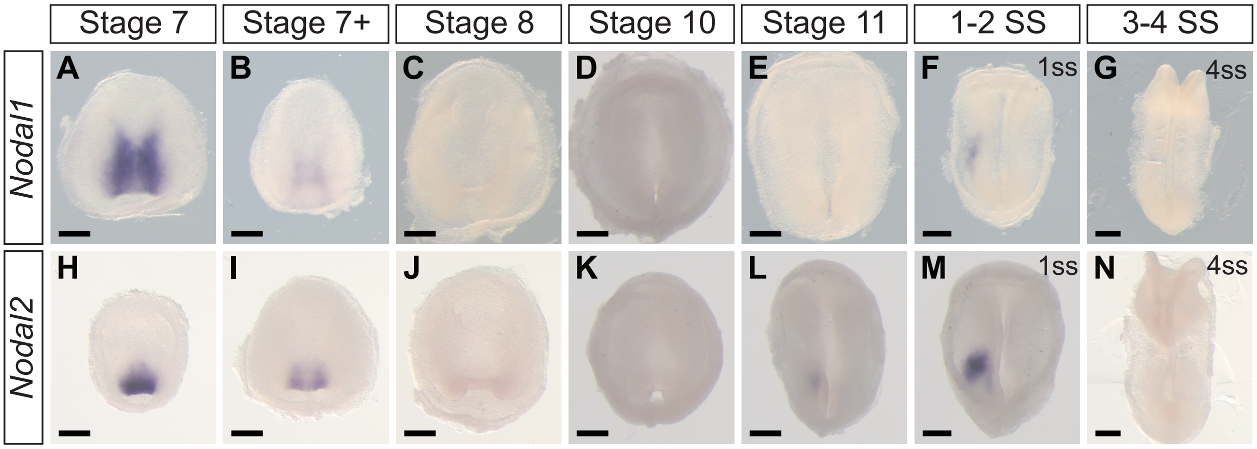


**Supplementary Figure S2**

Dorsal view of *Nodal1* and *Nodal2* expression. All embryos are presented in dorsal view. Ventral view is available in Figure 2. **(A-G)** Whole mount RNA *in situ* hybridization for *Nodal1* expression. **(H-N)** Whole mount RNA *in situ* hybridization for *Nodal2* expression. All scale bars are 200 µm.
