# Supplementary material for: Morphological changes and two Nodal paralogs drive left-right asymmetry in the squamate veiled chameleon (C. calyptratus)
Source: Front Cell Dev Biol. 2023 Apr 11;11:1132166. doi: 10.3389/fcell.2023.1132166 (PMC10126504; doi:10.3389/fcell.2023.1132166)
Supplement: Supplementary file 6 [file Image3.pdf]

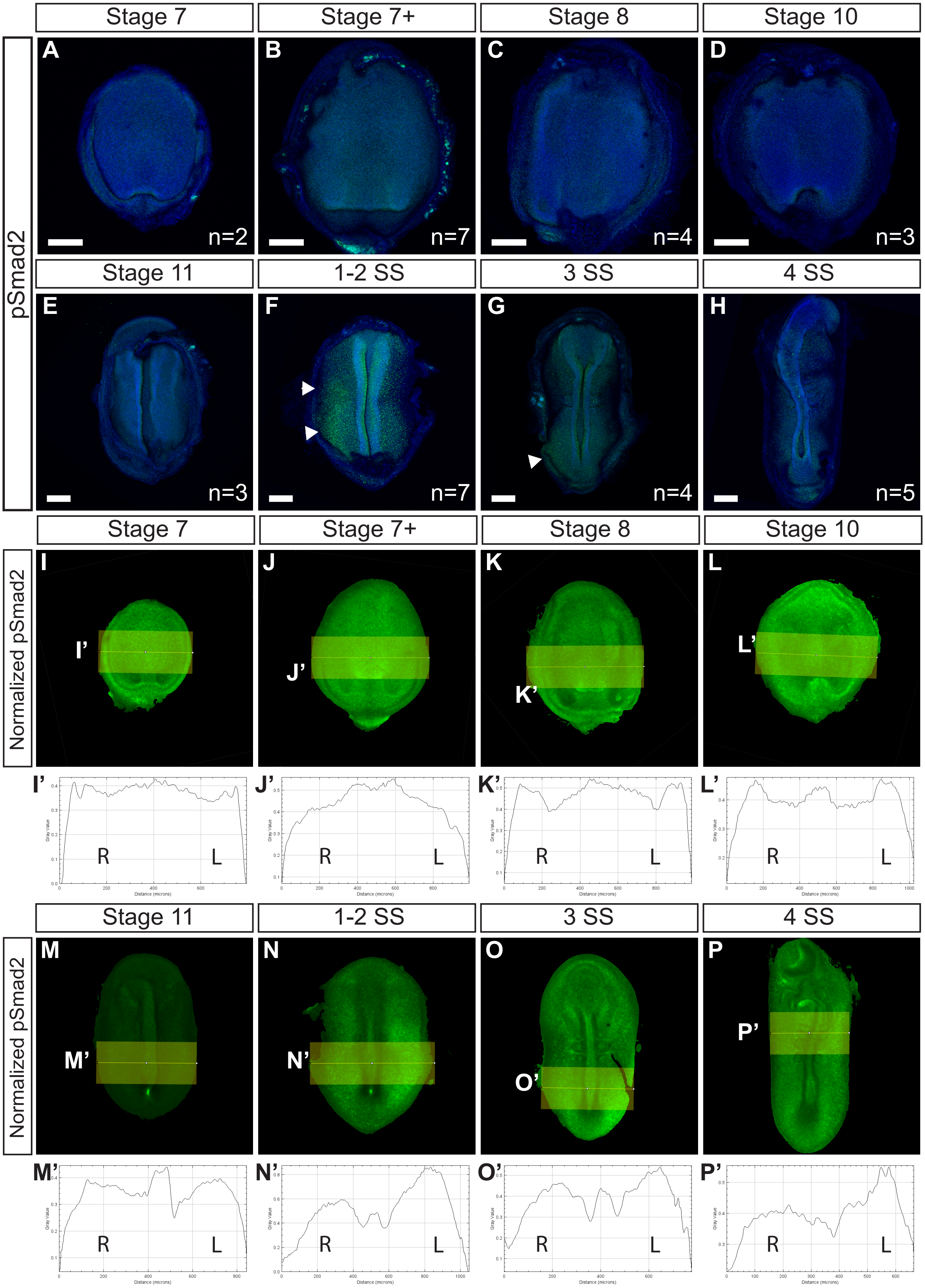

pSmad2
Normalized pSmad2
Stage 7
Stage 7+
Stage 8
Stage 10
Stage 11
1-2 SS
3 SS
4 SS
A
B
C
D
E
F
G
H
I
J
K
L
M
N
O
P
n=2
n=7
n=4
n=3
n=3
n=7
n=4
n=5
I'
J'
K'
L'
M'
N'
O'
P'
R
L
Gray Value
Distance (microns)

Supplementary Figure S3

pSmad2 staining reveals Nodal1 and Nodal2 activity in veiled chameleon embryos. Embryos in **(A-H)** are dorsal view, and embryos in **(I-P)** are ventral view of embryos in Figure 2 **(O-V)**. **(A-H)** The presence of active Nodal signaling was evaluated through antibody staining for pSmad2 (Green), with nuclear DAPI staining in blue. White arrowheads denote areas of pSmad2 enrichment visible in this view. **(I-P)** pSmad2 staining normalized to DAPI staining. **(I'-P')** intensity analysis between left and right sides on embryos, imaged in ventral view and normalized to DAPI. Areas analyzed as indicated in **(I-P)**. R (right) and L (left) denote the equivalent side of the embryo in the image analyzed. n indicates the number of embryos examined for a given stage. All scale bars are 200 μm.
