# Supplementary material for: Morphological changes and two Nodal paralogs drive left-right asymmetry in the squamate veiled chameleon (C. calyptratus)
Source: Front Cell Dev Biol. 2023 Apr 11;11:1132166. doi: 10.3389/fcell.2023.1132166 (PMC10126504; doi:10.3389/fcell.2023.1132166)
Supplement: Supplementary file 7 [file DataSheet1.pdf]

Supplementary Dataset 1. Embryo staging, based on (Dufaure and Hubert, 1961), adjusted for veiled chameleon-specific developmental features.

Stage (Dufaure and Hubert, 1961)	Description
Gastrulation	
7	The embryo is mostly circular. The blastopore forms a canal, with the posterior end elevated over the plane of the embryo on the ventral side. The ventral opening is to the anterior, appears concave and relatively narrow. The dorsal opening of the blastopore is also narrow. The dorsal blastoporal lip is concave, with the opening towards the posterior, reminiscent of letter "C".
7+	The embryo elongates slightly and appears ovoid. The blastopore opening broadens, and the posterior blastoporal lip becomes reminiscent of letter "M".
Neurulation	
8	Prechordal plate appears and can be distinguished as a slight anterior invagination on the ventral side. Ventrally, the blastopore has a wide concave rectangular opening towards the anterior. On the dorsal side the blastopore is concave, with the opening towards the posterior. The overlap between the ventral and dorsal lips of the blastopore narrows.
10	The blastopore opening has migrated and is open dorso-ventrally. The axial mesoderm is distinct, with mesodermal groove beginning to form. Early evidence for the formation of the head process and the foregut in the anterior.
11	The blastopore opening is closing and becomes reminiscent of a slit. The mesodermal groove is distinct. The head process and the foregut in the anterior are becoming more prominent.
16	2 somite pairs. The head process and the foregut in the anterior are becoming further distinct. On the dorsal side, neural folds are formed. The blastopore opening is closing, and the posterior tissue becomes elevated over the blastopore.
17	3 somite pairs. The foregut pocket and the head prominence are fully formed
18	4 somite pairs. The cephalic region is starting to tilt to embryo's right.

DUFAURE, J. P. & HUBERT, J. 1961. Table de développement du lézard vivipare: *Lacerta (Zootoca) vivipara* Jacquin. *Archives Anatomie Microscopie Morphologie Experimental*

50**,** 309-328.

Supplementary Dataset 2. Veiled chameleon RNA *in situ* hybridization probe sequences

>*Nodal1*
TCTGGCTGAGGTTCGACTTCACCTGCCCAGTTCTGGAGAGAAATCTACCTTTAACAG
TGCCCCAATGTGGGTAGACCTGTATCACCAGCAGGAAATCCATTGCTCAAGTGGTCA
CAACTGCCCACACATTGTTCACATAGGCTCTTTTGAAGCGCCTCCATCATTGCCTTCA
AGCTGGATAGTTCTGGAAGTCACGGAACAGCTCTCAAAGTGGGTAGTGAACAGCAG
CTTAGTGGAGGAATCTATTCAGAAAACTCTAAGTGAGGAACACCAGCATAGCAAAC
ATTTGCCAAAGCAAGCAACAAGTTACTGTGGATCTATAGATAGAAAGGCATTCTTAG
TGCTTTTCTCTAGACTTTCCAAAGAAGAGAAAGAAAGAAATAGCTCAACTTTGCTCC
AGATAGTGAAAGACTCAAAATATCTTACACTGAAGAATCCAAAAGATGTGGTACCT
GTTTCGGGAACAAAGAGGTATCGGCGCCACAAGAAACTCCAAGGCTGGTCTTTAAT
GAGTTTGAAAGAACTCAAGGTCGAAAACCTATGCCATAGAGTGGACTTCTATGTTGA
TTTTGAGGAGATAGGCTGGGGCTCCTGGATTATATACCCCAAGAGATATAATGCCTA
CCGCTGCGAGGGCATGTGTCCTA

>*Nodal2*
ACCTCGTCCTTCTCGTAGTAAAGCATGGAGAGTGGGCTCAATTTGACTGGAGCGCAG
GCTAGGCAAGGCACCCGGTTGGGTTGGTAGAACTTCAGCAAGCTCTGCATATATGCA
TGGTTGGTTGGCTTAAAGGTTTCATCCACAGGTGTTGGGCACTCCCCCTCACAACGG
TATGCATTATACTTCTTGGGATGTAGGATCCACATTCCAAAACCAGTCTTCTCAAAG
TCCACAATCATATCAACTCTCTTGCACAGGGATTTATTTTCTTCAGCAAAGCCAAGC
ACGTCAGCGTCGCTCCCTTTGATCCTTTTCCTTTGCTTCCTGTTCCGTCGGTGCCTTCG
GCCCCTGGGCTCTTTGTGTGCGTCATCTGACATGACGTATTTGGACATCTCTACCACC
CTGATGAGACTTGATCTCTGATAGGGTTCCATCTGTTCTTTATCTTTGGAGAAGATAA
CCAGAAGGACTTGTTCTTTCCTGCTCTGCAACGGTACTTCTGGGGTTGGACTGAAGT
CACAGATGTTCTCAAACCCATTGGTGGGGCATCCACTGGTTCCTTCGTCATTGCCATT
ATCTAGGCAATTTGCAGTCCATTCCTGCTCCTGAACATCTAGGGTATCGTGACCTGA
G

>*Lefty*
GTTTCCCTTCTTGACAAGGTACATCATGGGAAGTGGGGAACTTTCCACCACAGCACA
GTTTCTCTCTCCATAGCCAAAATGCCGCAACCAGTTCTTGGGTTGCCGGCAGCAACC
CATACAGTGGTAAGCCTGATAGCCAGCTGGCTCAATAATCCAATATTGAGTCCAAGT
TAACTCTCGGAAATTGATGTAATGTTCTTGCCGGCAGCAGGTGGTTTTGTCCGTTGAT
GCACCTTCTTTACAGTCTCCAGGTCCTCCATACTCTTCCAAGTTTATTGTATAAAGCA
CTAACTCAGGCTTGCCCAACGTCTTATCGGAGGGGTCCTGAGAAGTAAAGCGCACA
ACTCTGGCCATTTCTGAGGCATAGTTGCCTATTCTTTCTCCTTCAATCCAGACCTCCA
GAAGCATTGGCCCAGTTTTCTTCACTTTAAGCCAAAAATGTACAGCCTGGGTGACAT
CAAAATTCTTCCAGCCAGATTCCATAATTGGGACCAATCTGGAATCAATCAAAGTAG
TCCGGTTGGTTCCATTATCTCCAAGCTCTACCCAGTAGATGCTCACCCGGGCATTCA
AGACGGGTCTTTGAGATTGCCTGCTGGGCAGGTTTTTATATCCAGGGGCTTCTTGA
AAAGTTTCAGCTCAGCCAT

>*Cer1*
CTGCAAACATCCTGGTACATTTCATTGATCTTGATTGGAAGGACAATCTCTTCAGAC

CTTGATTTGGTTTTCAACATGAACAGATCCCAGAACTTCTTGGCATCTTTTCGGAAGA
CCAGGTTTGCCTTTCTCTTAGCGTGCTGATGCGGAGGATGGTTTGAGGGGGTTTCTG
GGTATGAAGGTGGATCTGTGGGAATCCAGCTTTCCAAATCCTGAGCCATATGGCCAC
CAACATGTGGGAAAATTGTCCTGGACACTTCAGGTTCATCTTGTGTCTTGCTCTCTGT
TTCTGCCAAGACAGCTGCCACAAACGGATCAGGCTGTGCCAGATACCTCACTAACA
AATCCTGAGGCAACTCCCGACTTTGTTCTGCTCTCTCAGGCCATGAAATCAGCAGCA
ACAAATACAAGATGCAGGCCTTG

>*Pitx2*
GGATCCGTCCAAGAAGAAGAGGCAGCGGCGGCAGCGCACGCACTTCACCAGCCAGC
AGCTCCAGGAGCTGGAAGCCACCTTCCAAAGGAACCGCTACCCGGACATGTCGACG
CGGGAGGAGATCGCCGTCTGGACCAACCTCACCGAGGCCCGAGTCAGGGTGTGGTT
CAAGAACCGCCGGGCCAAGTGGCGGAAGCGGGAACGGAACCAGCAGGCGGAGCTG
TGCAAGAACGGCTTCGGGCCGCAGTTCAACGGCCTGATGCAGCCTTACGACGACAT
GTACCCGGGCTACTCGTACAACAACTGGGCGGCCAAGGGCCTGACGTCGGCCTCGC
TCTCCACCAAGAGCTTCCCCTTCTTCAACTCCATGAACGTCAACCCGCTCTCGTCGCA
GAGCATGTTCTCGCCGCCCAACTCCATCTCGTCCATGAGCATGTCTTCCAGCATGGT
CCCCTCGGCCGTGGCCGGCGTCCCGGGCTCGGGCCTCAACAGCCTCAACAACTTGAA
CAACCTGAGCAACCCTTCGCTCAACTCGGCCGTGCCCACGCCCGCCTGCCCTTACGC
CCCGCCGACCCCTCCTTACGTTTACCGGGACACGTGTAACTCCAGCTTGGCCAGCCT
GAGACTCAAAGCCAAGCAGCACT

>*Shh* [1]
TCCGGCTTCGACTGGGTCTATTACGAGTCCAAGGCTCACATCCACTGCTCGGTCAAA
GCCGAGAACTCAGTGGCAGCCAAATCCGGTGGCTGTTTCCCCGGAACAGCCTGGGT
GAACCTGGAGCAAGGAGGAACCAAGCTGGTGAAGGACCTCAACCCTGGAGACCGG
GTTTTGGCAGCGGACACCCAGGGCCGCCTGCTCTTCAGTGAATTCCTCACCTTCCTG
GACCAAGAGGAGGCCCCGATCCACAAACTCTTCTATGTCATTGAGACCCAAAGGCC
CCGGACACGTCTCTTGCTGACGGCGGCCCACCTACTCTTTGTGGCCTCACCCCAGAA
CCAGTCTCAACCCCAGCCCATTTTTGCCAGCCGTGTACAGCCAGGACAACATATCTA
CGTGCTCAGCCAAGGAGGTCAGACACTGCTAGAAGCTGCAGTGCACCGGGTTTCCC
TGCAAGAGGAAGCTTTGGGGGCCTACGCCCCACTGACCGCCCATGA

>*Chordin*
CATACCCTATGGGAGAGATCCAAGGGAAGATTATCAAGCATCGGGCCCTCTTTGCA
GAAACATTCAGTGCCCTCCTGACATCTGCGGACCCAACCCACCTTGGCATGGGTGGC
ATTGCTATGCTTACCTTGAGTGACACAGAGAACAACCTACATTTTGTCCTAGTGACC
AGGGGGCTTTTGGAGCCAGCTGACAAAAAATCCTCCTCGCTCCCCTTGAGGGTACAA
ATCCTGCACCAGGACAGAGTTCTGAGGGAAATGCTTGCCAATGTTACCTTGCAGGCC
TCTGACTTCGCAGAAGTGCTGACTGGACTTGGAAGTGAAGAGATGCAGTGGCTGGC
CCACGGGGCATTGAGGATTACAGCAGAGGTGGAGGGGAAATTCAGGCGCCAGATTG
CTGGACAGATCACCCCAAGGAGAAGTTGTGACACCCTACAAAGTGTGCTTTGTGGA
GGAGATGCTTTGATACCGACCCAGACAG

>*Fgf8* [1]
TCTAGTGCGGACCTACCAGCTGTACGGTCGGACCAGTGGGAAGCACGTGCAAATTCT
GGACAACAAGAAAATCAATGCCTTGGCAGAGGATGGAGACGCTCACGCCAAGCTCA
TTGTGGAGACTGACACCTTTGGAAGCCGAGTAAGAATCAAGGGTGCTGAAACTGGC
TTCTATATCTGCATGAACAAGAAAGGAAAGCTGATTGGCAAGAGCAATGGCAAAGG
CAAGGACTGTGTCTTTACAGAGATTGTGTTGGAGAACAACCACACAGCACTGCAGA
ATGCCAAGTATGAAGGGTGGTACATGGCCTTCACCCGAAAGGGGCGCCCCCGCAAA
GGCTCCAAGACTCGTCAACATCAACGTGAGGTGCACTTCATGAAGCGCTTA

>*FoxJ1*
CATTAGGCACAACCTCTCCTTGAACAAGTGCTTCATCAAAGTCCCACGAGAGAAGG
ATGAACCAGGAAAAGGTGGCTTCTGGAAAATTGACCCTCAGTATGCTGACAGACTG
ATGAACGGGGCATTCAAGAAGCGCAGGATGCCTCCAGTGCAAATCCATCCGGCTTT
CAGTGGACGAATGCAACAGGATGCCTGCTCCAGTTCTTCTGCTCAGCAGGCTGCAAT
CTCTTGGAAAAACAATGGCATCCTAAAAATCAACATGGAGTCTCAGCAGCTACTCA
AAGAATTTGAAGAAGTCACTAGTAGTGATCAAAACTGGAATCCAGCAGTGGATGGG
AAAATGAGCCATAAACGTAAGCAGCCTTTGCCAAAACGGATGTACAAGACTGCCCG
CCTCTCCAGCTCTCCCATGCTGACACAGGAAGAACAAACAGAGCTTGGATCTCTGAA
AGGTGACTTTGATTGGGAAGCTATCTTTGACACCACTTTAAATGCTGATTTTTCCACC
TTTGAAGATTTGGAGATCACGCCTCCTATTAGCCCAATAACCAGGGATGTAGATTTG
ACTGTGCATGGAAGACATATCGATTGCCCACAGGAGTGGTGCCCCACTGGGCAGGA
TTACGTTCTAACAGAGTCCAACCAGAACAGCTTAGACTTTGATGAAACCTTCATTGC
TACTTCTTTCCTCCAGCATCC

>*Dnah11*
CTGCCAGTCTTTTTGAAGTGGTCAGTCCAGATTACAAACAACTGAAACAGTGTCGCA
AGGAAATAACATTGCTGAAGGGAATGTGGGACATCAATATTTATGCAACAAGTAAC
ATCAGTGATTGGATTAAAAGCCCTTGGAGGGAGATTAGTATGGAGCAGATGGATGC
AGAACTGAGAAGATTTGCAAAGGAGTTGTGGGCACTGGATAAAGAAGTTCGCTCCT
GGAATGTATACACAAATCTGGAACTAACAATTAAAAACTTGTTGACATCGTTGAAG
GTTGTTACAGAGTTGCAGAATCCAGCCATGAGGGACAGGCACTGGCATCAGCTGAT
GGATGCAATAGGTATTCAGTTTTCAATAAGTGAAGATACAACATTGGCAGATTTGTT
AGCGCTGAAGCTCCACAAGATGGAAGATGATGTCAGAAACATTGTTGACAAAGCAG
TAAAAGAACTTGGGATTGAAAAGATTCTCACAGAAATCAACCAAATATGGGCTACA
ATGGAGTTTTGTTATGAAGAGCATTACAGGACCAGTGTTCCTTTGTTGAAAACAGAT
GAGCACCTTTTTGAGACATTAGACGATAACCAAGTTCAGCTGCAAACAGTTCTGCAA
AGTAAATATGTTGAGTATTTCATTGAGCAAGTTTCAAACTGGCAAAAAAAGCTAAAT
ATTGCAGACTCTGTAATTTTTCTTTGGATGGAAGTTCAGCGCACATGGTCTCATCTTG
AAAGCATTTTCATTGG

1 Diaz, R. E., Jr. & Trainor, P. A. Hand/foot splitting and the 're-evolution' of mesopodial skeletal elements during the evolution and radiation of chameleons. *BMC Evol Biol* **15**, 184 (2015).
