# Supplementary material for: Morphological changes and two Nodal paralogs drive left-right asymmetry in the squamate veiled chameleon (C. calyptratus)
Source: Front Cell Dev Biol. 2023 Apr 11;11:1132166. doi: 10.3389/fcell.2023.1132166 (PMC10126504; doi:10.3389/fcell.2023.1132166)
Supplement: Supplementary file 11 [file Image1.pdf]

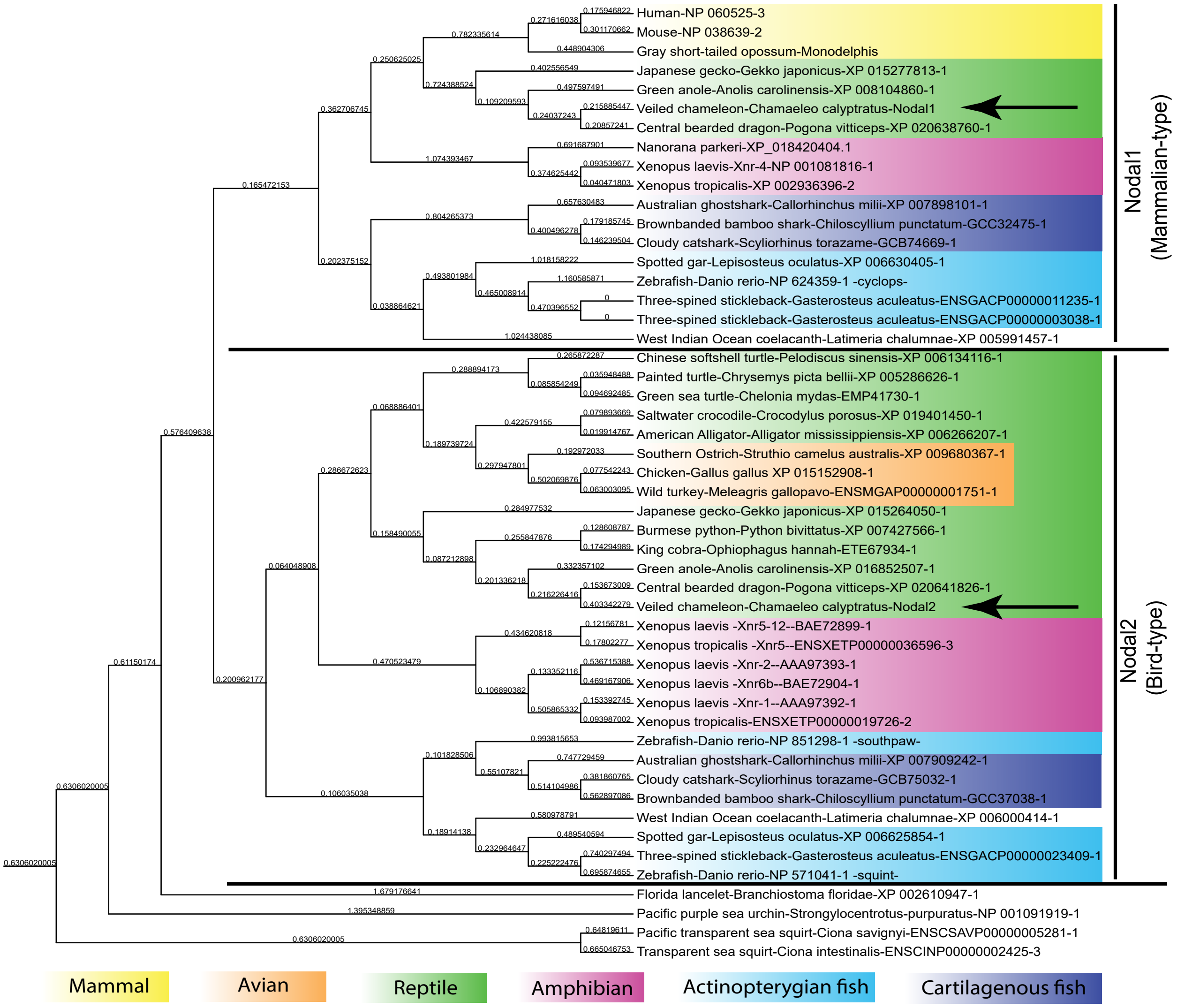

Supplementary Figure S1

Molecular phylogeny of Nodal proteins. Veiled chameleon possesses two different transcripts, closely related to *Nodal* genes. Of the two ORFs, one sequence most closely aligns with Nodal1/mammalian type, and the other – with Nodal2/bird type (arrows). Background colors indicate taxonomic groups, in which selected species are categorized, and follow annotation laid out in Kajikawa et al. (2020). Not all phylogenetic branches are consistent with known phylogenetic relationships between species, likely skewed by mutation rate and limited sequence input. The numerical values represent branch lengths (mutations per cite).
